# Supplementary material for: Confusion2Vec 2.0: Enriching ambiguous spoken language representations with subwords
Source: PLoS One. 2022 Mar 4;17(3):e0264488. doi: 10.1371/journal.pone.0264488 (PMC8896703; doi:10.1371/journal.pone.0264488)
Supplement: S1 Appendix — (PDF) [file pone.0264488.s001.pdf]

S1 Appendix.

Table 6. Results: Analogy Tasks.

	Model	Analogy Tasks			
		S&S	Acoustic	S&S-Acoustic	Average Accuracy
	Google W2V [4]	61.42% (69.1%)	0.9% (1.42%)	16.99% (26.42%)	26.44% (32.31%)
	In-domain W2V	59.17% (68.14%)	0.6% (0.96%)	8.15% (13.5%)	22.64% (27.53%)
	fastText [9]	**75.93%** (**84.85%**)	0.46% (0.65%)	17.40% (24.90%)	31.26% (36.8%)
	In-domain fastText	46.45% (55.42%)	0.75% (1.25%)	17.05% (20.70%)	21.42% (25.79%)
**Confusion2Vec 1.0** (word) [10]	C2V-1	61.13% (70.56%)	0.9% (1.46%)	16.66% (23.86%)	26.23% (31.96%)
	C2V-a	54.99% (63.97%)	9.04% (16.92%)	34.61% (43.34%)	32.88% (41.41%)
	C2V-c	52.4% (65.45%)	16.54% (27.33%)	27.46% (38.29%)	32.13% (43.69%)
**Confusion2Vec 2.0** (subword)	C2V-1	46.75% (56.83%)	0.96% (1.46%)	16.64% (20.99%)	21.45% (26.43%)
	C2V-a	48.99% (56.74%)	**34.84%** (**50.79%**)	**34.91%** (**44.67%**)	**39.58%** (**50.73%**)
	C2V-c	48.94% (56.87%)	**34.87%** (**51.00%**)	**35.12%** (**44.98%**)	**39.64%** (**50.95%**)

C2V-a: Intra-Confusion; C2V-c: Inter-Confusion; C2V-1: Top-Confusion; S&S: Semantic & Syntactic Analogy; UJO: Unrestricted Joint Optimization (see [10]). Numbers inside parenthesis are for top-2 evaluations; Numbers outside parenthesis are for top-1 evaluations. Note: The relative improvement from top-1 evaluation to top-2 evaluation for Confusion2vec is significantly higher than baseline models. Bold numeric correspond to the results outperforming Confusion2Vec 1.0 in each evaluation task.

Table 7. Results: Analogy Tasks (Concatenated Models).

	Model	Analogy Tasks			
		S&S	Acoustic	S&S-Acoustic	Average Accuracy
	Google W2V [4]	61.42% (69.1%)	0.9% (1.42%)	16.99% (26.42%)	26.44% (32.31%)
	In-domain W2V	59.17% (68.14%)	0.6% (0.96%)	8.15% (13.5%)	22.64% (27.53%)
	fastText [9]	**75.93%** (**84.85%**)	0.46% (0.65%)	17.40% (24.90%)	31.26% (36.8%)
	In-domain fastText	46.45% (55.42%)	0.75% (1.25%)	17.05% (20.70%)	21.42% (25.79%)
**Confusion2Vec 1.0** (word) [10]	C2V-1 + C2V-a	56.51% (67.03%)	13.59% (25.43%)	31.74% (40.36%)	33.95% (44.27%)
	C2V-1 + C2V-c	53.14% (70.84%)	20.99% (35.25%)	25.05% (35.18%)	33.06% (47.09%)
	C2V-1 + C2V-c (UJO)	50.68% (65.88%)	33.05% (**49.4%**)	29.35% (41.51%)	37.69% (**52.26%**)
**Confusion2Vec 2.0** (subword)	fastText + C2V-a	67.95% (**76.10%**)	10.69% (22.67%)	**39.29%** (**49.15%**)	**39.31%** (49.31%)
	fastText + C2V-c	67.55% (**76.16%**)	10.91% (22.56%)	**39.36%** (**49.12%**)	**39.27%** (49.12%)

C2V-a: Intra-Confusion; C2V-c: Inter-Confusion; C2V-1: Top-Confusion; S&S: Semantic & Syntactic Analogy; UJO: Unrestricted Joint Optimization (see [10]). Numbers inside parenthesis are for top-2 evaluations; Numbers outside parenthesis are for top-1 evaluations. Note: The relative improvement from top-1 evaluation to top-2 evaluation for Confusion2vec is significantly higher than baseline models. Bold numeric correspond to the results outperforming Confusion2Vec 1.0 in each evaluation task.

Table 8. Results: Similarity Tasks.

	Model	Similarity Tasks	
		Word Similarity	Acoustic Similarity
	Google W2V [4]	0.6893 (0.6430)	-0.3489 (-0.3056)
	In-domain W2V	0.4417 (0.4416)	-0.4377 (-0.4216)
	fastText [9]	**0.7361** (**0.6942**)	-0.3659 (-0.3599)
	In-domain fastText	0.3584 (0.3769)	0.2610 (0.2758)
**Confusion2Vec 1.0** (word) [10]	C2V-1	0.6036 (0.6430)	-0.4327 (-0.4151)
	C2V-a	0.5228 (0.5030)	0.6200 (0.6116)
	C2V-c	0.5798 (0.5475)	0.5825 (0.5716)
**Confusion2Vec 2.0** (subword)	C2V-1	0.3720 (0.3867)	0.3022 (0.3155)
	C2V-a	0.2929 (0.3181)	**0.8108** (**0.9253**)
	C2V-c	0.2893 (0.3174)	**0.8106** (**0.9254**)

Numbers inside parenthesis are Pearson correlation; Numbers outside parenthesis are Spearman correlation. Bold numeric correspond to the results outperforming Confusion2Vec 1.0 in each evaluation task.
Note: Pearson correlation is higher than Spearman correlation for Confusion2vec 2.0 - hinting at relatively stronger linearity at tails of the distribution than monotonicity at and around mean of the distribution.

Table 9. Results: Similarity Tasks (Concatenated Models).

	Model	Similarity Tasks	
		Word Similarity	Acoustic Similarity
	Google W2V [4]	**0.6893** (**0.6430**)	-0.3489 (-0.3056)
	In-domain W2V	0.4417 (0.4416)	-0.4377 (-0.4216)
	fastText [9]	**0.7361** (**0.6942**)	-0.3659 (-0.3599)
	In-domain fastText	0.3584 (0.3769)	0.2610 (0.2758)
**Confusion2Vec 1.0** (word) [10]	C2V-1 + C2V-a	0.5102 (0.4794)	0.7231 (0.7258)
	C2V-1 + C2V-c	0.5609 (0.5152)	0.6345 (0.6164)
	C2V-1 + C2V-c (UJO)	0.5379 (0.4954)	**0.7717** (0.7908)
**Confusion2Vec 2.0** (subword)	fastText + C2V-a	**0.5744** (**0.5428**)	**0.7577** (**0.8184**)
	fastText + C2V-c	**0.5732** (**0.5424**)	**0.7573** (**0.8184**)

Numbers inside parenthesis are Pearson correlation; Numbers outside parenthesis are Spearman correlation.
Bold numeric correspond to the results outperforming Confusion2Vec 1.0 in each evaluation task.
