# Supplementary material for: Confusion2Vec 2.0: Enriching ambiguous spoken language representations with subwords
Source: PLoS One. 2022 Mar 4;17(3):e0264488. doi: 10.1371/journal.pone.0264488 (PMC8896703; doi:10.1371/journal.pone.0264488)
Supplement: Attachment — Submitted filename: response_to_reviewers.pdf [file pone.0264488.s003.pdf]

Confusion2Vec 2.0: Enriching ambiguous spoken language representations with subwords

Response to Reviewers:

We would like to thank the editor and all the reviewers for their valuable time in reviewing our paper and offering thoughtful and constructive feedback. We have gone through the comments carefully and made our best efforts in addressing each point made by the reviewers. We have incorporated the suggested modifications, which we believe has helped increase the quality of the paper substantially. The comments of the reviewers are cited and the detailed responses (marked in blue) to them are presented below.

Reviewer Comments:

Reviewer 1:

This is a very interesting paper – a marriage between acoustic knowledge and language processing. I think the direction of this research is great, and you are harnessing knowledge from two systems that have been well-researched for a very long time. The paper is well-written, conducting analysis from different angles with adequate reasoning for various design choices. The details of the experiments are also provided, along with the code which makes the study reproducible. Especially, the visualisations are pretty cool, and the essence of what is being captured by the models is very clear. However, the paper needs to focus on a few more aspects like:

- the potential application of such embeddings where acoustically similar words have a similar feature space (a potential application where we can use these embeddings instead of an ASR).
- the resource requirement for these embeddings
- what information are the newly developed embeddings missing

I think the authors have this information already, but they just have not reported it. Below are my comments and revision points organised at the section level.

Abstract: The abstract is well-written and the contribution of the paper is clear. However, include a statistical evidence of the significantly better performance that is claimed eg: an xx% increase)

Thank you for the suggestion. We have added the statistics under abstract. The following modification has been made (9th line):
"The results significantly outperform existing word vector representations when evaluated on erroneous ASR outputs, providing improvements up-to 13.12% relative to previous state-of-the-art in intent detection on ATIS benchmark dataset."

Introduction:

1. *"Although, there have been few attempts in leveraging information present in word lattices and word confusion networks for several tasks"* – this sentence undermines the amount of work that has happened with word lattices and confusion networks. Even the references you have mentioned contain numerous citations. Rephrase the sentence clearly stating that the representations using lattices and confusions networks have been successful in multiple tasks, however, they have some limitations.

   As per your suggestion we have now rephrased the sentence as follows:

"Prior attempts at leveraging information present in word lattices and word confusion networks have been successful for multiple tasks [12-17]. However, they have some limitations, as these prior works estimate the embedding in a supervised manner specifically trained with task specific labels. Consequently, the main downside is that the word representation estimated by such techniques are task-dependent and are restricted to a particular domain and dataset."

2. The motivations need to be referenced. For example, sentences like:
    - the acoustically ambiguous words tend to have more similar bag-of-character n-grams
    - subwords help model under-represented words more efficiently
    - subwords enable representations for out-of-vocabulary words

   Thank you for the comments. We have now added appropriate citations. Also, the sentence "the acoustically ambiguous words tend to have more similar bag-of-character n-grams" is a consequence of how the subwords are generated.

3. Somewhere in the introduction the difference of this study from the initial Confusion2Vec model has to be clearly mentioned.

   We thank you for the comment. We have now made the distinction clear under Introduction, paragraph 6. The modification is as follows:
   "In this paper, we extend the previously proposed Confusion2Vec representation framework by incorporating subwords to represent each word for modeling both the acoustic ambiguity information and the contextual information."

4. *"the main downside with these works is that the word representation estimated by such techniques are task-dependent and are restricted to a particular domain and dataset."* – has this been experimentally verified? If yes, state the reference. If no, this sentence will have to be rephrased. If we have a large text database of a language (which often exists in atleast the well-resourced languages like US English) and a relatively smaller domain-specific text databases, the representations should still be good for the domain-specific task. As for the speech database, dealing with "unseen" words in ASRs is a problem that is more general than specific to this paper's theme.

   Thank you. We would like to point out and clarify that the cited prior works estimate the vector representations using task and domain dependent speech datasets. These prior works make use of task-specific supervised training with ASR lattices as inputs. For example, spoken language intent detection or slot-filling. The data limitations are resultant of two factors: (i) task specific labeled speech data, and (ii) lattices generated through ASR of the speech datasets. We realize that the above may not have been conveyed clearly. We have added the following text under Introduction, paragraph 5 to enhance the clarity:
   "Prior attempts at leveraging information present in word lattices and word confusion networks have been successful for multiple tasks [12-17]. However, they have some limitations, as these prior works estimate the embedding in a supervised manner specifically trained with task specific labels. Consequently, the main downside is that the word representation estimated by such techniques are task-dependent and are restricted to a particular domain and dataset."

Confusion2Vec:

- The section heading should be a bit more descriptive – maybe Confusion2Vec representation framework?

  Thank you for the suggestion. We have renamed the section heading to "Confusion2Vec Representation Framework".

Confusion2Vec 2.0 subword model

- *"We believe we have a compelling case for the use of subwords for representing the acoustic similarities (ambiguities) between the words in the language since more similarly sounding words often have highly overlapping subword representations."* – reference for this statement? More clearly, why do you think it's a good representation?

  Similarly sounding words have highly overlapping subword representations – this is a consequence of how the subwords are generated, i.e., character n-gram encoding. These subwords can approximate/capture syllables in the language. Since the similarly sounding words tend to have similar set of syllables, this leads to higher similarity in encoding of subwords. A feature capturing such information helps in modeling the ambiguity information. We have rephrased and added better description as follows:
  "We believe we have a compelling case for the use of subwords for representing the acoustic similarities (ambiguities) between the words in the language for the following reasons. Similar sounding words often tend to have similar set of characters and thus have a high degree of overlapping set of character n-grams. This subword based feature encoding helps model the level of overlap and estimate the magnitude of acoustic similarity robustly. Moreover, the use of subwords should help in efficient encoding of under-represented words in the language \cite{bojanowski2017enriching}. This is crucial in the case of Confusion2vec because we are restricted to speech data and their corresponding decoded ASR lattices for training which leads to data sparsity issues compared to computing typical word vector representation that can be trained on large amounts of easily available plain text data. Another important aspect is the ability to represent out-of-vocabulary words \cite{oovsubword} which are common with end-to-end ASR systems outputting character sequences."

- *"use of subwords should help in efficient encoding of under-represented words in the language."* – reason for this or reference?

  Thank you. We have added the appropriate reference to the above statement.

- *"In the proposed model, each word w is represented as a sum of its constituent n-gram character subwords."* – Replace with - "In the proposed model, for example, … "

  Thank you. This has been replaced.

- *"The n-grams are generated for n=3 up to n=6."* – Why? Why is n=3 and n=6 the maximum and minimum limits for English? Is this based on language analysis, if yes, provide references.

  Yes, indeed the n-gram character range is language dependent. The character n-gram range was chosen based on empirical evidence obtained from a prior work. We have now added the reference and modified the text as follows:
  "The choice of length of character n-grams is language dependent and empirically chosen for English \cite{bojanowski2017enriching}."

Training Loss and Objective

- Equation (4)'s description is ambiguous. Is the equation that of the binary logistic loss? It is the objective function for subword model's negative sampling. Please mention that clearly, and add an LHS to this function.

  We regret the lack of clarity. We have rephrased the description and included the LHS for the function.

The following changes have been made:
"The negative sampling loss function to be optimized for subword model can be expressed as:"

$$J(\theta) = log\ \sigma(\sum_{s \in S_{w_i}} x_s^T o_{w_t}) + \sum_{k=1}^{K} \mathbb{E}_{w_k \sim P_n(w)} log\ \sigma(-\sum_{s \in S_{w_i}} x_s^T o_{w_k})$$

- Are there any other differences between the new implementation and the old Confusion2vec? Other than that, you are using subwords here? Again, any other changes should be clearly mentioned.

Yes, practically, the major difference is use of subwords. The implications of use of subwords are multifold and has been discussed throughout the paper.

Evaluations:

- *"useful, meaningful information embedded in the word vector representation"* – what is the difference between useful and meaningful in this context? Can it be useful but not meaningful, or can it be meaningful but not useful?

Thank you for the question. The word "useful" is used in the context of the embedding providing performance benefits with respect to the task (from a machine's perspective). Whereas the word "meaningful" is from a human's perspective, more specifically in terms of visualizations.

- For all the databases you have used, clearly mention the language of the database, and size of the database. This is essential for someone trying this out in another language.

Thank you, we would like to clarify all the databases, evaluations are in English language. To make this clear, we have added the following text under Evaluation section (1st paragraph):
"Note, all the evaluations, analysis and databases used in this work are in the English language."

The database description under section "Analogy & Similarity Tasks", subsection "Database" is self-explanatory – "Fisher **English** Training corpus".

We have also added the information under section "Spoken Language Intent Detection", subsection "Database". The following modifications have been made:
*"The dataset consists of humans making flight-related inquiries in the **English language** with an automated answering machine with audio recorded and its transcripts manually annotated."*

We have mentioned the size of the database in Section "Analogy & Similarity Tasks" under "Database" and "Experimental Setup".

- W2V – first time usage needs to full form.

Thank you for pointing it out. We have now used the full form.

- You mention the Word Similarity task – did you use human annotators for this? Or did you just use the results from [20] – in either case that has to be mentioned clearly, including number of people who annotated.

The word similarity task uses the WordSim-353 database. This database consists of 353 word pairs which are human annotated on the perceived word similarity by the annotators. We make use of these human annotated scores and calculate the correlation against the cosine similarity obtained using the various embedding spaces. We have included better description for better clarity under

section “Evaluations”, subsection “Analogy and Similarity Tasks” (see here).

- A description of the results is needed. Did your evaluation show that Confusion2vec 2.0 is better or comparable to existing representations?

  Thank you for your comment. The description and discussion of the results are presented in the subsequent section “Analogy & Similarity Tasks” under subsection “Results”. The current section “Evaluations” is meant to discuss and describe the evaluation techniques adopted in our work.

- What do the bold numbers in the Appendix Table 5 mean?

Thank you. We have fixed any inconsistencies in bold numbers. We have now specified what the bold numbers mean under the caption of Table 6, 7, 8 and 9. *“The bold numeric correspond to the results outperforming the Confusion2Vec 1.0 in each evaluation task”.*

Analogy & Similarity Tasks

- Automatic speech recognition – how important is the performance of the ASR to the Confusion2Vec training? Will an ASR with better performance (better WER) be better for the Confusion2Vec training?

That is a good question. The effect of ASR performance on the quality of embeddings has not been investigated as part of this study. However, it is an interesting aspect to be investigated in the future. Generally, we believe, the ASR should have a reasonable performance. For example, we don’t want an ASR that makes too many errors which would result in too many conflicting words ending up in the confusion network, meanwhile, we also don’t prefer an ASR that is too accurate, since that would result in very few word confusion in the lattices. Moreover, we believe that there are many aspects to this question, for example, the beam width used during decoding could have an effect in addition to the word error rate, furthermore, different noise/channel conditions, like reverberation could pose different dimension to this investigation. Overall, the answer to the posed question is not trivial and needs extensive investigation over multiple aspects effecting ASR performance. Hence, we leave this to future work.
We have added a brief discussion regarding this to the future work under the section Conclusion (4th paragraph, last 2 lines):
“We also plan to understand the factors that affect the quality of the proposed embeddings by conducting extensive analysis of the effects of ASR performance (WER), decoding beam size, characteristics of underlying speech signal environments including type of noise, amount of noise, channel effects, transferability over different ASR systems etc. The performance implications of these factors to the end-task are also of interest.”

- *“Also, a minimum frequency threshold of is set and the rarely occurring words are pruned from the vocabulary.”* – what is the motivation for this other than reducing the training time/resources? Will Confusion2Vec representation be able to deal with “unseen” words?

Thank you for your comment. Setting a minimum frequency threshold to prune rarely seen words is a standard practice for training most word vector representations. The reasoning behind this is that sparsely available words result in inaccurate representation due to poor estimation of the underlying distribution. Too few occurrences of a word results in erratic vector updates (insufficient statistics for reliable estimates). Pruning such words have been proven to result in more robust estimation and accelerates learning [1][2].

We would further like to clarify that our word vector representation has the ability to deal with unseen words. In case of unseen words, the vector sum of its constituent subwords is computed and used as the word vector representation of the unseen word. We have demonstrated this by performing specifically designed analysis by visualizing the embedding for the out-of-vocabulary word "prinz". Please see the discussion presented under section "Analogy & Similarity Tasks", subsection "Embedding Visualization".

[1] Mikolov, T., Sutskever, I., Chen, K., Corrado, G. S., & Dean, J. (2013). Distributed representations of words and phrases and their compositionality. In Advances in neural information processing systems (pp. 3111-3119).
[2] Bojanowski, P., Grave, E., Joulin, A., & Mikolov, T. (2017). Enriching word vectors with subword information. Transactions of the Association for Computational Linguistics, 5, 135-146.

- Under the results section – are the analogy and similarity tasks performed on the 353 pairs? Please refer to the relevant section.

We regret the lack of clarity regarding the analogy and similarity tasks. We have added better description of these evaluation databases and re-structured the content under section "Evaluations", subsection "Analogy and Similarity Tasks" to enhance readability. We have also listed the number of analogy questions present in each analogy-based tasks and the number of word pairs for the similarity tasks. The modified descriptions are listed below for your convenience:

"Analogy and Similarity Tasks:

For evaluating the inherent semantic and syntactic knowledge of the word embeddings, we employ two tasks: (i) the semantic-syntactic analogy task, and (ii) the word similarity task.

Semantic-Syntactic Analogy Task: *The word analogy task was first proposed in \cite{mikolov2013efficient} which comprises word pair analogy questions of the form W_1 is to W_2 as W_3 is to W_4. The analogy is answered correctly if vec(W_1) - vec(W_2) + vec(W_3) is most similar to vec(W_4). The task comprises 19,544 analogy questions as originally compiled and released by \cite{mikolov2013efficient}. We prune the analogy question database to match the training dataset vocabulary and to obtain identical setup used in \cite{shivakumar2019confusion2vec} for comparison purposes.*

Word Similarity Task: *Another prominent approach in the NLP community \cite{pennington2014glove,schnabel2015evaluation} for evaluating word vector representations is the word similarity task. We use the WordSim-353 database \cite{finkelstein2001placing} consisting of 353 pairs of words manually annotated over a score of 1 to 10 depending on the magnitude of word similarity as perceived by humans. The task involves computing the rank-correlation (Spearman correlation) between the human annotated scores and the cosine similarity of the corresponding word vector pairs \cite{schnabel2015evaluation}. A high correlation indicates that the word vector representation captures the word similarity order similar to that perceived by humans.*

For assessing the word acoustic ambiguity (similarity) information, we conduct the Acoustic analogy task, Semantic&syntactic–acoustic analogy task and Acoustic similarity tasks, all proposed in \cite{shivakumar2019confusion2vec}.

Acoustic Analogy Task: *The Acoustic analogy task comprises word pair analogies compiled using homophones which answer questions of the form: W_1 sounds similar to W_2 as W_3 sounds similar to W_4. The task comprises 2,678 analogy questions and is designed to assess the ambiguity information embedded in the word vector space \cite{shivakumar2019confusion2vec}.*

Semantic&Syntactic-Analogy Task: *The semantic&syntactic-acoustic analogy task is designed to assess semantic, syntactic and acoustic ambiguity information simultaneously. The analogies are formed by replacing certain words by their homophone alternatives in the original semantic and*

syntactic analogy task \cite{shivakumar2019confusion2vec}. The task comprises 3860 analogy questions. Examples of the analogies can be found in \cite{shivakumar2019confusion2vec}.
Acoustic Word Similarity Task: *The acoustic word similarity task is analogous to the word similarity task, i.e., it contains 943 word pairs which are rated on their acoustic similarity based on the normalized phone edit distances. A value of 1.0 refers to two words sounding identical and 0.0 refers to the word pairs being acoustically dissimilar. The task involves computing the rank-correlation (Spearman correlation) between the normalized phone edit distances and the cosine similarity of the corresponding word vector pairs."*

- Why do you think Confusion2Vec 2.0 performance is lower compared to Confusion2Vec and FastText for S&S analogy task?

Thank you for the question. We believe that two factors result in slightly lower performance on S&S analogy task in case of both Confusion2Vec and Confusion2Vec-2.0 compared to fastText:
1. Modeling: The additional acoustic ambiguity information that is being modeled in case of Confusion2vec can be considered nearly orthogonal to the semantics/syntax of the language. This makes any performance improvements among the ambiguity dimension result in slight degradation on the other dimension (semantics and syntax) inevitable. We believe the challenge is to obtain better trade-offs with respect to the end-tasks.
2. Evaluation: The analogy tasks are scored only if the most-similar word is the correct answer. Although such an approach seems fair in case of testing the contextual relations (Semantics and Syntax) in a language, the scheme is not optimal when testing for inter-relations across two disconnected dimensions (acoustic ambiguity and Semantics/syntax). Even though, we have tried to address the evaluation up to an extent by introducing top-2 evaluations for analogy tasks in case of Confusion2vec, there is a possibility that the embedding space prioritizes certain information dimension in special cases.

We have added the following discussion under the section "Analogy & Similarity Tasks", subsection "Results" (3rd paragraph, last but 2nd sentence).
"One explanation for this is that the different analogy tasks are fairly, mutually exclusive, i.e., getting right on one task compromises performance on the other. The top-2 evaluations for Confusion2Vec provides a partial solution to this. Nevertheless, there can be instances where the embedding can favor information on either acoustic ambiguity or contextual information dimension. Thus, there exists trade-off between the different proposed analogy based evaluation tasks. The goal is to optimize this trade-off as best as possible. One way to judge this trade-off is to look at the average accuracy across the analogy tasks."

- *"Investigating the results for the similarity tasks, we find a significant correlation of …"* – how was this correlation calculated? Did you have annotators perform the task for you? Or used the results from past annotations?

Apologies for the lack of clarity. We have added more detailed description of the evaluation tasks (see here) including the similarity tasks under section "Evaluations", subsection "Analogy and Similarity Tasks".

Model Concatenation

- *"The subword models slightly under-perform in the acoustic analogy task …"* This is a very interesting result and contradictory to what we expect. Why do you think this is the case? It feels that in these concatenations, the impact of fastText is dominant than Confusion2Vec.

Thank you. As discussed before, this is again a consequence of the trade-off between modeling acoustic ambiguity and contextual information associated within a language. The emphasis is to

optimize this trade-off in favor of end-task performance. Please note, in case of concatenations of two vector spaces, we are optimizing a totally different criterion opposed to the model without concatenation. We empirically find that concatenating models favor semantic and syntactic relations and also enhance the semantic&syntactic-acoustic dynamics. We have added the following text under subsection "Model Concatenation", (3rd paragraph, last line):
"Overall, these changes in dynamics between the acoustic and semantic/syntactic subspaces observed in the case of concatenated models can be attributed to the fact that we are optimizing a different criterion than the non-concatenated versions."

- *T*his is a general comment for all the training you have mentioned in this paper. To allow resproducibility of your results, and to allow other researchers to judge whether the resources they have are sufficient to undergo your experiments – please provide details of your computational resources and the training time needed. This maybe a separate section, or even included in Appendices.

  Thank you for the suggestion. We have added the following information under Section "Analogy & Similarity Tasks", subsection "Experimental Setup", under "Confusion2Vec 2.0":
  "The models were trained on CPU only with the following machine configuration: dual Intel Xeon CPU E5-2670 operating at 2.6GHz based on 64bit architecture. The machine had a total of 32 threads, i.e., each CPU comprises 8 cores with 2 threads per core. The machine was equipped with 128 GB of DDR3 memory, i.e., 8 x 8GB memory per CPU. With the above machine configuration and the above mentioned experimental setup, training intra-confusion model took approximately 46 minutes and inter-confusion model took approximately 3 hours and 24 minutes."

Embedding Visualization

- Give details of the packages used for the visualisation.

Thank you for the suggestion. The following details have been added in the 2nd line.
"The visualizations are generated using scikit-learn and matplotlib python packages."

- The example about "prinz" is interesting – but is this a one-off example? Are there other occurrences of words that are clustered together due to their acoustic similarity? Also, was prinz part of the training set?

The example word-pairs used in the visualizations are picked randomly but, in a way, to represent semantic, syntactic and acoustic relations. Please note, the visualizations are provided to humanly relate to the complex interactions of the acoustic and contextual subspaces. It is practically exhaustive to check for every such combinations visually. The analogy based evaluations as well as the word similarity evaluations are designed to check for such relations in a more practically feasible way. Thus, it is likely we will find many more such examples.

More examples of the acoustically similar words clustered together can be found in our previous publication on Confusion2Vec-1.0 (see Figure 12). Moreover, the acoustic analogy task and acoustic similarity task results also support the evidence.

The word "prinz" is out-of-vocabulary, meaning that it is not a part of the training set. The subword encoding enables to derive vector representations for such unseen words by computing the vector sum of its constituent character n-grams. We have mentioned this in the last line of the subsection "Embedding Visualization".

- It would be interesting to see a similar visualisation of Confusion2Vec 1.0 and the concatenated model too so that a comparison can be drawn with Confusino2Vec 2.0. I had a look at the

Confusion2Vec 1.0 paper, but, as the same word list is not used, a direct comparison is not possible.

Thank you for the suggestion. We would like to point out that similar visualization with identical word list for Confusion2Vec 1.0 and Google W2V can be found in our previous work – "Prashanth Gurunath Shivakumar, Mu Yang, Panayiotis G. Georgiou: Spoken Language Intent Detection Using Confusion2Vec. INTERSPEECH 2019: 819-823". We have now added the following text under Fig 2 captions to redirect interested readers to our previous work:
"Plots with identical word sets corresponding to Confusion2Vec 1.0 and Google W2V can be found in \cite{shivakumar2019spoken}. Please note that the out-of-vocabulary word `prinz' cannot be represented in Google W2V and Confusion2Vec 1.0 spaces."

We would like to emphasize that the visualizations provide the overall gist of the word spaces and should not be used to judge the performance differences between the vector spaces. For visualization purposes, we perform extreme dimension reduction to enable plotting vectors – which results in a lot of information loss compared to the original embeddings. For performance evaluations, the various analogy and similarity based tasks serve as indicators.

For your reference, we have included the plot with the concatenated model below:

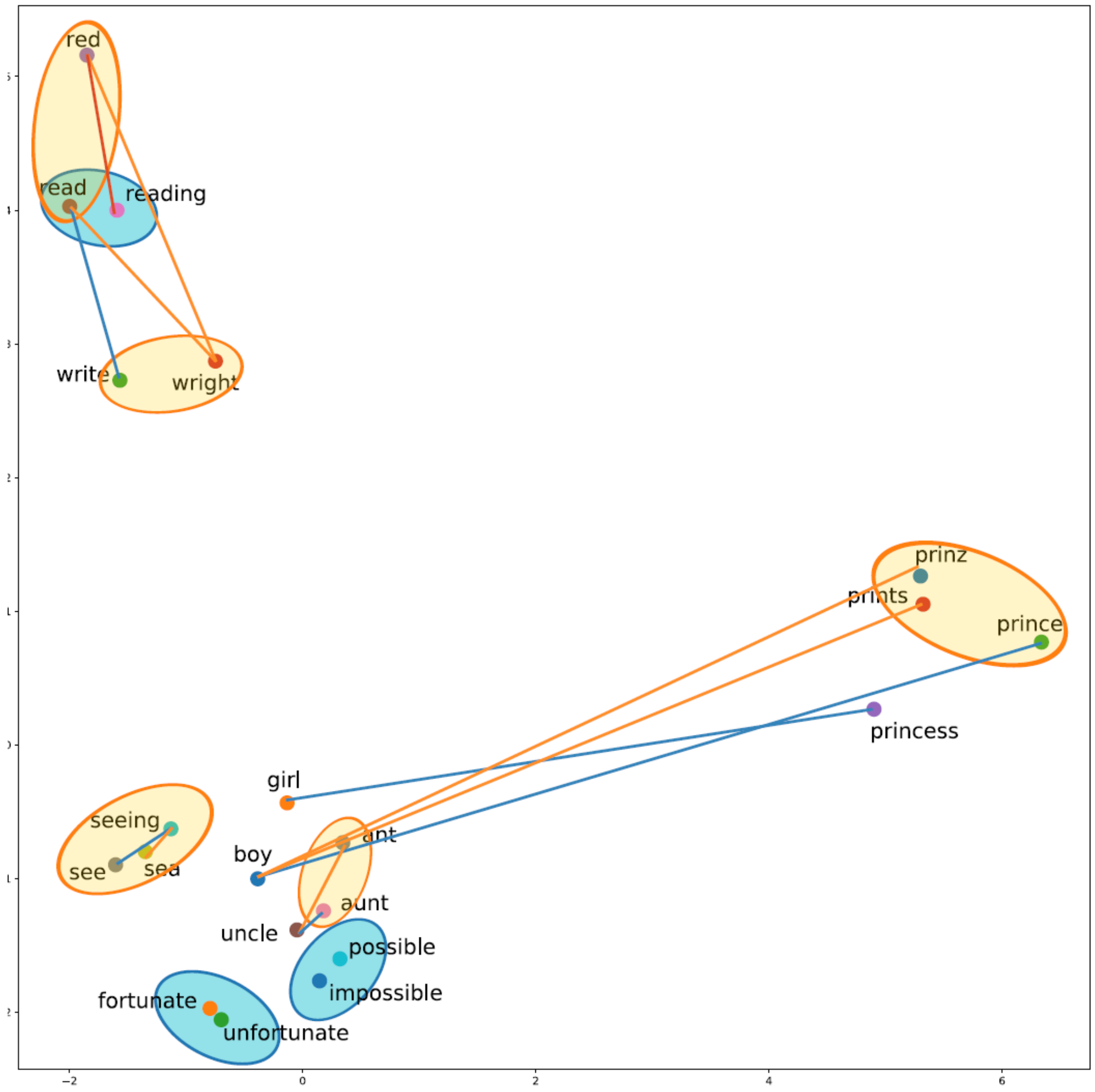

The visualizations corresponding to vector space plot of concatenated and non-concatenated versions of Confusion2Vec 2.0 are alike. Since both the version of the models are based on the same concept of joint modeling of ambiguity and context, we expect the plots to be similar. The main difference between the concatenated and non-concatenated versions are performance based, i.e., the concatenated version achieves a better balance of the two information dimensions. We skip the plot since it doesn't add to the paper.

- The visualization is interesting and gives a clear picture (literally) of what the models are doing. We can see that the Confusion2Vec 2.0 is clearly modelling human perception. But it feels like it is modelling human perception of *individual words in isolation* without the context. That would describe why it has such a close feature space for "prints" and "prince". But then, is that good? Do we not want our NLP applications to be able to differentiate these two words rather than consider them as *similar?* I think this also explains the high correlation you have got in the acoustic similarity tasks – basically where humans are finding individual words acoustically similar, Confusion2Vec 2.0 is also finding the same, and not otherwise. This needs to be addressed in your discussion:

  - Why is the Confusion2Vec in its embeddings training not capturing the context information? Or rather what can we do to make it capture context information AND acoustic similarity? Maybe the concatenated model is the solution for this. We can know this only by having a look at the visualisation.

  Thank you for your comments. The results from the semantic and syntactic analogy tasks, from Table 1, are evidence for the fact that Confusion2Vec is capturing context information. Please note that these analogy tasks are quite strict in assessments, i.e., any random embedding not capturing context information would give near 0% in semantic & syntactic analogy tasks. We agree that the performance is slightly less than embeddings trained solely on contextual information (word2vec and fastText) mainly because of reasons discussed earlier. Also, please note that, under Table 1, it is only fair to compare the "S&S" Analogy Task results of Confusion2Vec with the "In-domain" versions of fastText and Google W2V since the Confusion2Vec is trained only using "In-domain" data. This comparison further shows that the loss in contextual information is minimal.

  Moreover, there is evidence that Confusion2Vec captures context information even in the visualized plots. Please refer to our previous work - "Shivakumar, P. G., & Georgiou, P. (2019). Confusion2Vec: towards enriching vector space word representations with representational ambiguities. PeerJ Computer Science, 5, e195" for plots portraying exclusive semantic, syntactic word relations (Figures 7, 8, 9 and 10). We show that the Confusion2vec preserves the context information and augments acoustic ambiguity information efficiently. This is also the case with Confusion2Vec 2.0.

  - In what application would you want acoustically similar words to have a similar feature space? I understand it is good for cases like a noisy ASR output or mispronounced words.

  We believe any application involving speech signal (spoken language) should benefit with inherent acoustic ambiguity information embedded in word vector representations. For example, ASR, Spoken Language Understanding, speech translation, text-to-speech systems etc.. We agree that purely NLP based applications (with no ambiguity) may not benefit. However, given the evidence (see Table 3, comparing results under "Reference" column) that Confusion2Vec doesn't degrade performance in purely NLP applications as well (since it effectively preserves and captures similar context information as other popular alternatives such as fastText, Word2vec + additionally augments information that can potential provide benefits in different scenarios), there is no reason to discount the

Confusion2Vec in most NLP applications.

Moreover, the ambiguity need not be limited to acoustics only. Inherent ambiguities are present in various other scenarios dependent on the nature of underlying signals. For example, pictorial ambiguities associated in applications such as Optical character recognition or Image/Video Scene summarization. There is also possibility of multiple ambiguity dimensions associated with certain applications such as Speech Translation where in addition to acoustic ambiguity, there can be ambiguity associated with source-target language morphology, segmentation and paraphrases. More applications are discussed in detail in our previous work, please see section "Potential Applications" in ""Shivakumar, P. G., & Georgiou, P. (2019). Confusion2Vec: towards enriching vector space word representations with representational ambiguities. PeerJ Computer Science, 5, e195"

The following text has been added under the conclusion section (3rd paragraph) , discussing potential future applications:

"The proposed Confusion2Vec word embedding can benefit any application involving speech signal (spoken language) in which acoustic ambiguity is inherent, for example in scenarios involving ASR, error correction systems, spoken language understanding, speech translation, text-to-speech systems etc. Moreover, the ambiguity need not be limited to acoustics only. Inherent ambiguities are present in various other settings dependent on the nature of the underlying signals such as for example, pictorial ambiguities associated with applications such as Optical character recognition or Image/Video Scene summarization. There is also the possibility of multiple ambiguity dimensions associated with certain applications such as Speech Translation where in addition to acoustic ambiguity, there can be ambiguity associated with source and target language morphology, segmentation and linguistic expressions such as paraphrasing. More applications are discussed in detail in \cite{shivakumar2019confusion2vec}."

- Finally, what impact did the sub-word model bring here that a word-based model could not?

We have found that the sub-word modeling overall enhances the modeling capabilities of acoustic ambiguities. We obtain higher performance in both of the evaluation tasks. The analogy tasks and similarity tasks. More crucially, we observe significant improvements over word-based model in application to real world spoken language intent detection. The subword model also comes with certain additional perks of being able to represent out-of-vocabulary words.

it has such a close feature space for "prints" and "prince". But then, is that good? Do we not want our NLP applications to be able to differentiate these two words rather than consider them as *similar?*

Thank you for the comments. The experimental results presented in our previous works as well as the current paper indicates that the Confusion2Vec augments typical context based word vector representations with additional useful information such as any ambiguities that may be present in human spoken language or any other signal modalities. In other words, the Confusion2Vec is providing "additional" information that acoustically word "prints" sounds similar to word "prince" while retaining the contextual information. The Confusion2vec comprises of two principal subspaces, one comprising contextual information (similar to fastText/word2vecc) and another comprising acoustic signature information. Hence, depending on the scope of end-task

applications, the back-end classification models can choose to use any combination of the subspaces. For example, a purely NLP application may use just the contextual subspace and ignore the acoustic ambiguities, whereas any spoken language application may take into account crucial acoustic signatures of the words in addition to the contextual information.

Spoken Language Intent Detection

- In the Database section – what does "samples" mean? Sentences?

That's right. Samples in the context of ATIS dataset correspond to one sentence with an associated intent label.

- *"Among the different versions of the proposed subword based Confusion2vec, we find that the concatenated versions are slightly better."* – It does not look like they are "slightly" better, it looks like they are clearly better. Again, I think the visualisation of the concatenated models in the visualisation section is essential.

Thank you for the suggestion. We have now modified the text to indicate the concatenated version is clearly better. The modification is as follows:
"Among the different versions of the proposed subword based Confusion2vec, we find that the concatenated versions are better."

- Please provide some examples of the Intent detection task – sentences, along with human-annotated intent and ASR identified intent.

Thank you for the suggestion. We have added a table listing a few examples through the process of intent detection. The relevant discussion has also been added under Section "Spoken Language Intent Detection", subsection "Results" under "Training on Clean Transcripts", last paragraph. For your reference the Table and the discussions are given below:

Table 4. Examples of Intent Detection: Trained on clean reference text, evaluated on ASR transcripts.

System	Text	True Intent	Predicted Intent	
			fastText	concat C2V-c 2.0
Manual<br>ASR	"what is the seating capacity of a DC9"<br>"what is **to feeding** capacity of the DC9"	Capacity	Meal	Capacity
Manual<br>ASR	"what is the lowest fare for a flight from washington dc to boston"<br>"what is the lowest **fair** for a flight from washington dc to boston"	Airfare	Flight	Airfare
Manual<br>ASR	"list fares from washington dc to boston"<br>"**lift affairs** from washington dc to boston"	Airfare	Flight	Flight
Manual<br>ASR	"what does fare code bh mean"<br>"**which is could be <unk> me**"	Abbreviation	Ground Service	Abbreviation

Manual refers to clean, human annotated transcripts. ASR refers to the automatic speech transcription by ASR. The bold text highlights the errors made by ASR. "concat C2V-c 2.0" refers to the proposed model: concatenated fastText + inter-confusion model

"Further, analyzing the results, Table 4 lists a few examples within the domain of intent detection comparing the baseline fastText embedding and the proposed concatenated version of inter-confusion model. In the first example, the ASR incorrectly recognizes ``seating'' as ``feeding'' which leads to an error in intent classification, i.e., intent is detected as ``Meal'' instead of ``Flight Capacity''. However, Confusion2Vec is able to recognize the ambiguity through better vector representation of acoustic confusions between the two unvoiced fricatives /f/ and /s/ and the consonants /d/ and /t/, phenomena that are well documented \cite{kong2014classification,phatak2007consonant}, and eventually lead to better classification. The second example is a classic instance of homophones (fare and fair) with similar implications. In the third example, both the embeddings fail to recover from the error. Finally, the fourth example is a manifestation of a more complex error spanning words/phrases. The proposed Confusion2Vec is able to reconcile the acoustic ambiguity information across multiple

words and successfully recognize the correct underlying intent."

- *"This confirms our initial hypothesis that the subword encoding is better able to represent the acoustic ambiguities in the human language."* – are we sure that this experiment is proving that? The statement is ambiguous because it feels like the model is able to differentiate the ambiguous words – rather from the visualisation we see that it is clustering the ambiguous words together. Hence, this claim has to be made unambiguous. Also, the results are good for this particular task, or in tasks were its okay to have similar representation for ambiguous words. What about applications where a differentiation is needed?

Thank you for the comments. Please note in the visualization, in case of Confusion2vec 2.0 (Figure 2b), the model is not just blatantly clustering acoustically ambiguous words together. Instead, it is clustering the acoustically ambiguous words together while also attaching the semantic context to the acoustic alternatives. For example, the vector "boy-prince" is similar to "boy-prints" (cosine similarity). Also, the vector "boy-prince" is similar to vector "boy-prinz".

In application to the particular task, the following is a fair explanation:
For recovering errors made by the ASR, the backend intent classification model needs to know which set of words are acoustically ambiguous and in turn realize the most probable correct word given the context. For example, consider the true sentence "List all the flights flying today". Let's assume that the ASR makes an error as follows "List all the lights flying today". A typical word embedding modeling only context information can provide alternatives to the wrongly recognized word "light" which are semantically/syntactically close such as "shine", "fire", "sun" "illuminate". A word embedding modeling only the acoustic similarity can provide the erroneously recognized word "lights" with several acoustically ambiguous alternatives such as "slights", "plights" and "flights". However, Confusion2Vec can provide the correct alternative word, i.e., "flights", which is not only acoustically similar but fits in the context. Note, providing acoustic alternatives such as "slights" or "plights" and ignoring context information could instead confuse and deteriorate the performance.
Our representation does not blatantly cause more confusions in vector representations, but instead provide additional useful information. This is supported by the fact that the Confusion2Vec provides decent, comparable performance to popular word embeddings in tasks comprising clean transcripts and no errors (see Table 2). Benefits are evident when there are errors from ASR (see Table 3).

- *"These results prove that the subword-Confusion2vec models can eliminate the need for re-training natural language understanding and processing algorithms on ASR transcripts for robust performance."* – again too generalized – this is an intent classification task and the experiment only proves the efficacy of the model for this task or similar ones. It should not be generalized.

Thank you for the suggestion. We have rephrased the sentence to make it more specific to the task presented in the paper. The modified text is as follows:
*"These results demonstrate that the subword-Confusion2vec models can eliminate the need for re-training the **intent classification model** on ASR transcripts for robust performance."*

Conclusion

- A discussion section needs to be added that discusses the impact of the findings of this paper. A few points that can be discussed about are:
  - The impact of having context information for the Confusion2Vec embeddings.

    Thank you. We would like to clarify that we have demonstrated throughout our paper that the Confusion2Vec comprises context information.

○ Some applications of Confusion2Vec 2.0 – like what is the use of clustering similar sounding words together for an NLP application – especially without context information.

Thank you for the suggestion. We have now added possible applications of Confusion2Vec in NLP/SLU domains and implications of ambiguity modeling in other digital signal processing domains.

"The proposed Confusion2Vec word embedding can benefit a range of applications involving speech (spoken language) in which acoustic ambiguity is inherent, for example in scenarios involving ASR, error correction systems, spoken language understanding, speech translation, text-to-speech systems etc. Moreover, the ambiguity need not be limited to acoustics only. Inherent ambiguities are present in various other settings dependent on the nature of the underlying signals such as for example, pictorial ambiguities associated with applications such as Optical character recognition or Image/Video Scene summarization. There is also the possibility of multiple ambiguity dimensions associated with certain applications such as Speech Translation where in addition to acoustic ambiguity, there can be ambiguity associated with source and target language morphology, segmentation and linguistic expressions such as paraphrasing. More applications are discussed in detail in \cite{shivakumar2019confusion2vec}."

○ The resource requirement for this embeddings development – For some languages it may not be possible to have a large, annotated speech database for ASR modelling. So, it is important to state how much speech and text data is required.

Thank you for the suggestion. We haven't conducted any specifically designed experiments in this study regarding resource requirements. The consensus for training word embeddings in the NLP community is: "More the data better the embedding". This should also apply in our case. However, we would to point out that subword encoding allows for relatively better, robust embeddings for a given amount of data (especially in low data scenarios). Moreover, the unsupervised modeling and domain independent representation of Confusion2Vec, allows training on easily available, large amounts of speech data for applications towards any other domains. We have added the following statements under Conclusion (2nd paragraph) to highlight the strengths of the proposed embeddings:

"Further, the following advantages highlight the prospects of the proposed Confusion2Vec embedding in enabling its applications in a wide range of conditions: (i) the proposed Confusion2Vec embeddings provide feasible representations both acoustically and semantically for unseen and out-of-vocabulary words, (ii) the embedding is able to model domain independent representations in an unsupervised manner that can capture acoustic signatures of words in conjunction with semantic information, which enhances the flexibility and feasibility to train on easily available domain independent speech data, and (iii) the domain independent nature of Confusion2Vec enables cross-lingual modeling, transfer learning techniques \cite{adms2017cross,zoph2016transfer} for capturing ambiguous information in low-resource languages."

Reviewer 2:

The article proposed a Confusion2Vec 2.0 to handle the ambiguities found in natural language using subword modeling units. The article presents the performance over various evaluations tasks including

word analogy and word similarity tasks, which deal with acoustic, syntactic, and semantic ambiguities. The empirical evaluations presented in the article are thorough and have significant improvements over the existing methods.
Overall, the research article is mostly clear when it comes to related literature, methodology, and result analysis. The language is simple enough to read and understand. However, there are few flaws and questions throughout this article that authors should consider and clarify, and are mentioned below:

1. There are many state-of-the-art end-to-end ASR models exist today, why the traditional HMM-DNN based pipeline has been used?

Thank you for the question. Our choice of ASR was to match the setup of our previous published works to facilitate direct comparisons. This enables us to assess the impact of subword encoding. For your reference the following are the previous studies:

- Shivakumar, P. G., & Georgiou, P. (2019). Confusion2Vec: towards enriching vector space word representations with representational ambiguities. PeerJ Computer Science, 5, e195
- Prashanth Gurunath Shivakumar, Mu Yang, Panayiotis G. Georgiou: Spoken Language Intent Detection Using Confusion2Vec. INTERSPEECH 2019: 819-823.

However, we would like to clarify that this study can be replicated by more recent end-to-end ASR systems. Moreover, we want to emphasize that employing state-of-the-art need not necessarily improve the quality of the Confusion2Vec embedding. While a poorly performing ASR with a high WER is not preferrable (leads to too many, acoustically unrelated confusions), we also don't need a near perfect ASR since it might lead to too fewer acoustic confusions for the sake of training Confusion2Vec. We realize that it is an interesting question to know what WER bands are ideal for training Confusion2Vec. Also, we believe that there are many aspects to this question, for example, the beam width used during decoding could have an effect in addition to the word error rate, furthermore, different noise/channel conditions, like reverberation could pose different dimension to this investigation. Overall, further investigation is needed over multiple aspects effecting ASR performance. Hence, we leave this to future work.
We have added a brief discussion regarding this to the future work under the section Conclusion (4th paragraph, last 2 lines):
"We also plan to understand the factors that affect the quality of the proposed embeddings by conducting further analysis of the effects of ASR WER, decoding beam size, characteristics of underlying speech signal environments including type of noise, amount of noise, channel effects, transferability over different ASR systems etc. The performance implications of these factors to the end-task are also of interest."

2. Have you considered other modeling configurations other than inter and intra-confusion?

Thank you for the question. In our previous paper (Shivakumar, P. G., & Georgiou, P. (2019). Confusion2Vec: towards enriching vector space word representations with representational ambiguities. PeerJ Computer Science, 5, e195), we proposed 4 different configurations (i) top-confusion training, (ii) intra-confusion training, (iii) inter-confusion training, and (iv) hybrid intra-inter confusion training. Based on the findings regarding the effectiveness and quality of the Confusion2Vec embeddings, we narrowed our choice to mainly inter-confusion and intra-confusion configurations in the current paper.

3. The table captions should include the metrics of the results. For Eg. From table #1 and #2, it is tough to figure out what kind of results are presented.

Thank you for the suggestion. We have added the metric information to the captions under Table 1 and 2. For your reference, the following text has been added:
"The results of the analogy tasks represent percentage accuracy; and the results of the similarity tasks represent Spearman correlation."

4. There are no red lines and ellipses in Figure 2. I believe It should be orange.

Thank you for pointing this out. We have corrected and replaced "red with "orange" under caption of Figure 2.

5. There are many grammatical errors in the article. The examples can be found in lines #279 and #313, where "are" should be "is". Further, line #312 "is" should be "are".

Thank you for pointing out the grammatical errors. We have fixed the same. We have also gone through the entire paper to fix any additional errors to the best of our efforts.

6. There is some unnecessary hyphenation. Eg. Common-place (commonplace), hyper-parameters (hyperparameters).

Thank you. We have fixed these and any other occurrences in the paper.

7. Acronyms in the reference section should be in uppercase letters. Eg. Bert (BERT), Rnn (RNN), Asr (ASR), Glove(GloVe), and so on.

Thank you. We have gone through the entire reference section and fixed any such occurrences.

8. Missing full stop on line #194.

Thank you for pointing this out. We have fixed it.

9. The English article should be used wherever possible.

We added "the" at several locations where it was missing. Thank you for pointing this out.
